# Paving the Way toward Personalized Medicine: Current Advances and Challenges in Multi-OMICS Approach in Autism Spectrum Disorder for Biomarkers Discovery and Patient Stratification

**DOI:** 10.3390/jpm11010041

**Published:** 2021-01-13

**Authors:** Areej G. Mesleh, Sara A. Abdulla, Omar El-Agnaf

**Affiliations:** 1 Division of Genomics and Precision Medicine (GPM), College of Health & Life Sciences (CHLS), Hamad Bin Khalifa University (HBKU), Doha 34110, Qatar; Armesleh@hbku.edu.qa; 2 Neurological Disorder Center, Qatar Biomedical Research Institute (QBRI), HBKU, Doha 34110, Qatar

**Keywords:** autism spectrum disorder, biomarker, omics, precision medicine, proteomics, transcriptomics, epigenetics, metabolomics, patient stratification

## Abstract

Autism spectrum disorder (ASD) is a multifactorial neurodevelopmental disorder characterized by impairments in two main areas: social/communication skills and repetitive behavioral patterns. The prevalence of ASD has increased in the past two decades, however, it is not known whether the evident rise in ASD prevalence is due to changes in diagnostic criteria or an actual increase in ASD cases. Due to the complexity and heterogeneity of ASD, symptoms vary in severity and may be accompanied by comorbidities such as epilepsy, attention deficit hyperactivity disorder (ADHD), and gastrointestinal (GI) disorders. Identifying biomarkers of ASD is not only crucial to understanding the biological characteristics of the disorder, but also as a detection tool for its early screening. Hence, this review gives an insight into the main areas of ASD biomarker research that show promising findings. Finally, it covers success stories that highlight the importance of precision medicine and the current challenges in ASD biomarker discovery studies.

## 1. Introduction

Autism spectrum disorder (ASD) is a multifactorial neurodevelopmental disorder characterized by impairments in two main areas: social communication, which includes poor eye contact, difficulty understanding facial expressions, and delayed speaking skills; and repetitive and restricted behavior patterns such as hands flapping, headbanging, and complex body movements. ASD can also include individuals with intact language but impaired social and communication skills. The prevalence of ASD has increased in the past two decades [1]. However, it is not known whether the evident escalation in ASD prevalence is due to changes in diagnostic criteria or an actual increase in ASD cases. The prevalence of ASD varies. It accounts for 1–2.5% of the total population; also, males are more likely to be affected by ASD compared to females at approximately a 4:1 ratio [1,2]. Autistic disorder, Asperger’s syndrome (AS), childhood disintegrative disorder, and pervasive developmental disorder not otherwise specified (PDD-NOS) are classified under the umbrella of ASD. These sub-classifications are diagnosed using the criteria of the diagnostic and statistical manual of mental disorders (DSM-5) that was released by the American Psychiatric Association (www.dsm5.org) and the International Classification of Disease 10 (ICD-10). Other diagnostic tools such as Autism Diagnostic Observation Schedule (ADOS) and Childhood Autism Rating Scale (CARS) have further been used to diagnose ASD. In the majority of cases, ASD is diagnosed at school age [3], with a mean age of 6 years old [4], although it should be diagnosed earlier. Additionally, late positive diagnosis of ASD after an initial negative diagnosis is not uncommon, which may imply flaws in diagnostic methods [5,6]. Furthermore, due to the complexity and heterogeneity of ASD, symptoms vary in severity and may be accompanied by comorbidities such as epilepsy, attention deficit hyperactivity disorder (ADHD), and gastrointestinal (GI) disorders [7]. Moreover, given the lack of effective drugs to alleviate or reduce the core symptoms of ASD, early behavioral interventions are key for better outcomes, as it improves cognitive performance as well as behavioral and language skills [8]. Consequently, identifying biomarkers of ASD is not only crucial towards understanding the biological characteristics of the disorder, but also as a detection tool for its early screening. Additionally, it supports the currently available diagnostic methods and paves a platform for a more robust and objective methodology.

This review provides insight into the main areas of ASD biomarker research that show promising findings, especially in genomics, transcriptomics, proteomics, metabolomics, microbiome, brain imaging, and eye-tracking. Furthermore, this review discusses some success stories that show the importance of precision medicine and the current challenges in ASD biomarker discovery studies and patient stratification.

## 2. Genetics of ASD: Understanding the Etiology and the Heritability

Genetics studies account for the majority of research published on ASD [9]. The recurrence rate of ASD is 2–8% higher between the siblings of a diagnosed child compared to the general population. Moreover, the concordance of ASD in monozygotic twins (MZ) ranges between 60–92% and from 0–10% in dizygotic twins (DZ) [10]. These observations highlight the importance of genetic factors for diagnosing and understanding ASD pathogenesis. While identifying ASD causal genes may not change current intervention protocols, it would, however, help in understanding the etiology behind ASD and it may help in developing genetic biomarker diagnostic tools. In some cases, ASD manifestations have been linked to a variety of well-known single-gene (monogenic) conditions. Common examples are fragile X syndrome (FXS) caused by a mutation in the *FMR1* gene and tuberous sclerosis complex (TSC) caused by a mutation in *TSC1* and *TSC2* genes. The notion that ASD is not caused by a single gene can be deduced from the fact that the aforementioned conditions are caused by different genes and patients with these different conditions can develop ASD. Epidemiological studies found that 25–40% of individuals with the abovementioned conditions tend to develop autistic traits [11,12]. Interestingly, even though ASD is known to be highly heritable, the diagnostic yield of ASD in terms of genetic evaluation varies, as it reached 40% [13], and as low as 8–10% in other studies [14,15]. However, much of these findings depend on the diagnostic tiers and techniques implemented by the clinical laboratory. Moreover, only 5–15% of total ASD cases are attributed to the abovementioned monogenic conditions (i.e., FXS and TSC) [16]. Consequently, the majority of the cases are classified as idiopathic (iASD).

ASD can be caused by rare or de novo single nucleotide variants (SNV), structural variants (SV), or copy number variants (CNV) that may affect multiple genes [16,17]. Large SVs are linked to ASD along with other comorbidities such as epilepsy, hyperactivity, behavioral problems, schizophrenia, and dysmorphic features [18]. SVs are usually detected using cytogenetic and comprehensive genomic hybridization (CGH) techniques in addition to next-generation sequencing (NGS). The CGH technique enhanced the detection of SVs such as large and small/submicroscopic duplications deletions and inversions in non-syndromic autism and mental retardation conditions [14]. A genome-wide CNV study done on ASD patients and healthy controls showed that SVs tend to encompass *NLGN1, ASTN2, UBE3A, PARK2, RFWD2*, and *FBXO40* in ASD patients [19]. These genes are known to be involved in cell-adhesion and ubiquitin pathways, as these pathways are important for synaptic formation, neuronal connection, and proper neuronal cell functions as shown in Table 1.

On the contrary, common SNVs were also investigated in the context of ASD. As it is suggested that the majority of ASD cases are caused by common variants. These variants and their corresponding genes have different levels of penetrance that are known to impact chronic and complex diseases such as type 2 diabetes mellitus (T2DM) and cardiovascular diseases (CVD), as reported in many genome-wide association studies (GWAS). Furthermore, these diseases are similar to ASD in the sense that they all share a complex interplay between genetic and environmental factors, as incomplete penetrance of common SNVs in different genes contributes to the burden of these diseases/disorders along with lifestyle and other environmental factors. Similarly, GWAS and other genomic studies resulted in unwinding the complexity of ASD by identifying common variants associated with ASD. For instance, one GWAS study showed that certain SNVs that encompass genes such as *NEGRI, PTBP2, CADP2, KCNQ2, KMT2E,* and *MACROD2* significantly present in ASD subjects [22]. Nevertheless, according to the SFARI (Simon Foundation Autism Research Initiative), 913 genes are implicated in autism in humans (https://www.sfari.org/resource/sfari-gene/); some of these genes (Table 1) are important for brain development, synapses formation, and gene expression.

Overall, genetic and genomic studies are essential in understanding ASD etiology and pattern of ASD heritability, as well as family counseling, although there are some cases where the counseling recommendations are already fairly clear, such as for FXS. However, they may not be suitable as biomarker screening tools because of their complexity in terms of incomplete penetrance, high variability, polygenicity, and pleiotropic effect associated with ASD-related genes. Nevertheless, the implementation of sequencing technologies such as targeted sequencing, whole-genome sequencing (WGS), and whole-exome sequencing (WES) is expected to enhance the clinical diagnostic yield. Although NGS is still difficult to implement in clinical settings, a workflow that considers clinical manifestations and incorporates a variety of molecular techniques is suggested to reduce the cost and enhance the efficiency of ASD diagnosis.

## 3. Non-Coding RNA’s as Biomarkers for ASD

MicroRNAs (miRNAs) are a group of non-coding RNAs (ncRNA) family. Other forms of non-coding RNA include long non-coding RNAs (lncRNA), small nuclear RNA (snRNA), small nucleolar RNA (snoRNA), ribosomal RNA (rRNA), and pseudogenes. The main function of miRNA is to regulate gene expression at the posttranscriptional level. Since the discovery of ncRNA in blood and other body fluids, miRNAs in particular have gained considerable interest as potential biomarkers for diseases such as cancer [26]. A study examining the serum of ASD patients and matched controls, through the use of a miRNA PCR array specific for neurological miRNAs, identified five potential microRNAs that were capable of differentiating healthy subjects from ASD subjects; these miRNAs are miR-19b-3p, miR-130a-3p, miR-181b-5p, miR-320a, and miR-572 [27]. Additionally, others have shown that miR-140-3p was upregulated in both serum and saliva [28,29]. Interestingly, in these studies, miR-140-3p was detected in different techniques, RNA-Seq in saliva and TaqMan low-density array (TLDA) in serum which suggests that miR-140-3p might have a particular role in ASD. In line with the aforementioned studies, one study used a multiplex reverse transcriptase-polymerase chain reaction (RT-PCR) on ASD postpartum samples and found that miR-140-3p and other miRNAs’ expression were dysregulated in ASD cerebral cortex [30]. Furthermore, a large-scale study done on ASD patients used salivary miRNA to differentiate between ASD and other neurodevelopmental disorders such as developmental delay (DD) from typically developed children (TD). In this study, they found that the salivary miRNAs were able to differentiate ASD subjects with moderate accuracy [31]. Salloum-Asfar, Satheesh, and Abdulla have extensively reviewed ASD miRNA studies; these studies are summarized in Table 1 in their paper [32].

miRNA biomarker studies need further validation using in vivo and in vitro studies as the majority of existing studies used in silico prediction as a way to investigate the function and the validity of miRNAs discovered. Furthermore, inconsistencies between the studies in terms of findings do not necessarily mean that miRNAs fail as biomarkers, but rather, it may suggest two main issues: variation in protocols and the need for a better approach for ASD sub-classification. Although miRNA biomarkers have been the most popular in the ncRNA family, other ncRNAs have shown considerable potential as biomarkers. For instance, a study utilizing peripheral blood identified a signature set of 20 ncRNA, which includes some pseudogenes, lncRNA, snRNA, snoRNA, and rRNA. In their study, they discovered ncRNAs markers that showed an excellent robustness assessed by ROC curve analysis when tested on different ages, genders, and ASD sub-classifications [33]. Some of these ncRNA, such as POLR2KP2, TUBB2BP1, RNU1-16P, and RNVU1-15, are moderately to highly expressed in the neurons, which may suggest a possible association of these ncRNA to ASD pathogenesis in the brain. However, these findings need to be validated on a larger scale, because this study used a total of 186 samples divided into a training set and a validation set followed by 23 ASD patients and 23 controls samples to further validate these markers. Furthermore, the abovementioned studies underline the importance of utilizing panels that contain a group of a validated set of ncRNAs that could serve a purpose in discriminating ASD from normal children.

## 4. Evidence of Epigenetics Modifications in ASD

Epigenetic modifications, such as changes in DNA methylation patterns, are also elements that may potentially serve as biomarkers. As mentioned above, ASD widely overlaps with some monogenic conditions such as FXS and Rett syndrome. The pathophysiology of these conditions is known to be linked to dysregulation in DNA methylation [34,35]. A recent epigenome-wide DNA methylation study (EWAS), done on 223 postpartum brain sections of the prefrontal cortex, temporal cortex, and cerebellum, taken from 43 ASD patients and 38 non-psychiatric controls, showed significant differences in CpG methylation patterns; mainly in the cortical regions compared to the cerebellum in ASD brain tissue [36]. They inferred from their findings that there is a convergence in molecular signature between different forms of ASD [36]. Indeed, brain tissue is not accessible for biomarker discovery purposes; however, replication of these finds can be very powerful in validating the authenticity of the identified biomarker, as brain tissue is the main site of ASD’s pathophysiology. As a consequence, a study that was done on ASD peripheral blood showed that there is an overlap in methylation patterns between ASD and other mendelian neurodevelopmental diseases that display mutation in epigenetic machinery genes [37]. Although the overlap was minimal, it did suggest similarities in the initial events of these disorders. Moreover, their machine-learning tools were able to differentiate the unique epi-signature of each disorder, which is crucial for diagnosing diseases with overlapping clinical manifestations [37].

A study done on ASD discordant twins using lymphoblastoid cells derived from blood lymphocytes showed that 2 CpG islands were hypermethylated. These CpG islands belong to B-cell lymphoma 2 (*BCL2*) and RAR-related orphan receptor A(*RORA*) genes, their findings were confirmed using bisulfite sequencing and methylation-PCR, and these genes were found to be downregulated in the brain [38]. Moreover, a study done on ASD MZ pairs using whole-peripheral blood revealed differences in methylation levels at many CpG sites. Moreover, they found that these changes in DNA methylation surround genes that have been previously linked to ASD, such as AF4/FMR2 family member 2 (*AFF2*)*, NRXN1, NLGN3,* and *UBE3A* [39]. On the other hand, a large-scale case-control EWAS used DNA from blood failed to attribute any CpG site to ASD because they did not achieve the Bonferroni discovery threshold (*p* < 1.12 × 10^−7^) [40].

The contradictions between these findings suggest that the sample of choice, and maybe the cell type/matrix used may affect the final results. Unlike genetic mutations, epigenetic modification such as methylation is tissue-specific, and using different cell types/matrix or even a mix of a heterogeneous population of cells may influence the findings. Another essential point is the selection of the studied subjects. For instance, as mentioned above, Nguyen et al. [38] and C. C. Wong et al. [39] studies used ASD twins, as these subjects share genetics, age, and the maternal environment in utero. However, in the Andrews et al. study [40], although they used a considerably large sample size, the heterogeneity of their ASD subjects may have hindered possible CpG methylated sites; unlike the abovementioned studies that used twins, who are more likely to share many of the genetic and environmental exposures.

## 5. Proteomics: A Fundamental Tool That Helps in Biomarker Discovery

Proteins are the final products that carry function. Protein abnormalities reflect upstream molecular problems that occur at the DNA and the RNA levels and these problems can be mirrored by a change in protein activity, structure, and abundance. Similarly, proteins can also be modified by external stimuli. For example, high sugar intake will increase glycated hemoglobin 1c (HbA 1c), and this biomarker is important for long-term monitoring of a diabetic’s diet [41]. There are many different methods of using proteomics for biomarker discovery studies. Common, unbiased approaches include a bottom-up approach, where the proteins are digested into peptide fragments and then run into mass spectrometry (MS) [42], and a top-down approach, where the intact proteins are separated and run through MS. Both strategies allow for gel and gel-free separation of the proteins/peptides [43]. Moreover, the top-down approach is better at identifying post-translational modifications (PTM) [43]. Applying the MS-based proteomics approach for biomarker discovery has opened the door for neurodevelopmental and neuropsychiatric disorders providing an unbiased method for a better understanding of these complex conditions. Other methods of proteomic biomarker investigation are immunoassays. These methods require prior knowledge of the protein’s function and their expression in a particular tissue/body fluid. Consequently, this allows for the generation of immunoassays to capture proteins and evaluate possible upregulation/downregulation as well as PTM on a set of proteins. These techniques are used mostly in the validation phase after biomarker discovery phase and they are easy to implement in a clinical setting [44].

Proteomic investigations on the gray matter of the frontal lobe of ASD individuals, identified single amino acid substitutions from alanine to glutamic acid in a protein called glyoxalase I (Glo1). This noticeable change has a higher frequency in ASD postpartum brain tissue compared to healthy controls [45]. This substitution caused a reduction in the enzymatic activity of Glo1 which, as a consequence, resulted in the accumulation of advanced glycation end products (AGE). Therefore, Glo1 substitution may affect certain crucial functions during neural development in early life [45]. Furthermore, a study exploring the Brodmann area 10 (BA10) and the cerebellum region (CB) of postpartum tissues showed differential expression of proteins related to synaptic connectivity, axon myelination, glial cells function, and metabolic activities in both regions [46]. Some of these proteins are glial fibrillary acidic protein (GFAP), creatine kinase B (CKB), myelin basic protein (MBP), and synapsin II (SYN2).

**Figure 1:**
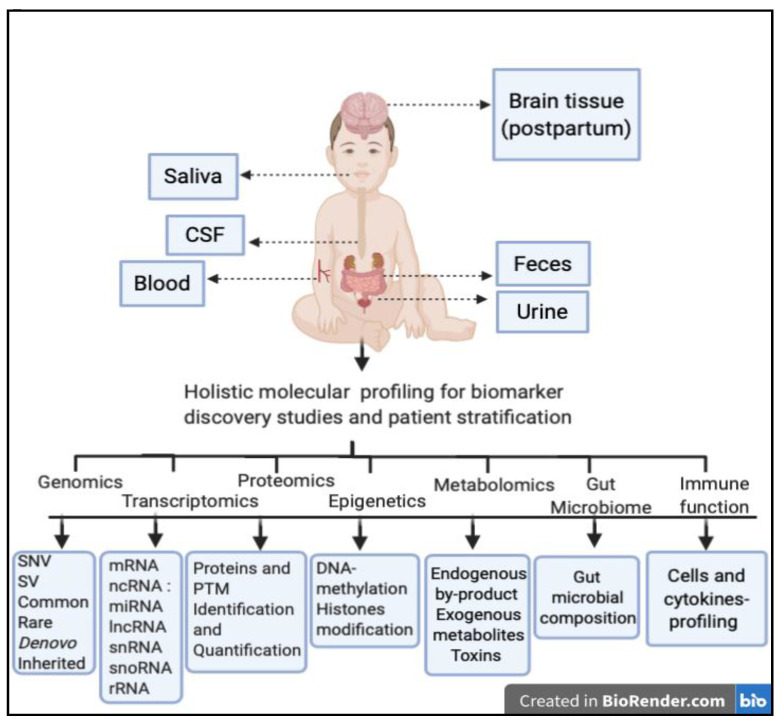
Schematic showing possible body fluids and tissues that may be essential for biomarker discovery in ASD patients. Blood is the most common site for biomarker discovery. However, saliva, urine, and feces are easily accessible and have been used recently for biomarker discovery studies. Although CSF and brain tissues could also be used for biomarker discovery in ASD, their accessibility is very difficult or even impossible (in the case of the brain, unless it is postpartum) for such purposes. Collecting huge biological data from a range of body fluids may help in performing holistic molecular profiling in the area of genomics, transcriptomics, proteomics, epigenetics, metabolomics, gut microbiome, and immune system, which may enhance biomarker discovery and patient stratification.

Many studies tried to identify biomarkers in accessible body fluids such as cerebrospinal fluid (CSF), serum/plasma, saliva, and urine, Figure 1. For instance, a quasi-prospective study was done on neonatal CSF samples, that were formerly collected from mildly febrile neonates (0–3 months old) that later developed ASD, to check whether biochemical differences exist before ASD is phenotypically manifested. They showed that arginine vasopressin (AVP) is significantly decreased in 11 ASD neonates compared to 22 controls [47]. This study was based on a pre-determined knowledge about the role of AVP and preliminary results published earlier [48]. The same group had found reduced CSF AVP levels in ASD’s samples and lower levels were associated with severe ASD symptoms. Unlike the former study, this study focused on older 1.5–9 years old ASD subjects [49]. Both studies imply a persistent and robust link between AVP and ASD even at an early age, as early as a few days. Interestingly, no significant difference was found on oxytocin (OXT) between the groups [47]. These findings may underline a possible subtype of ASD with a dysregulation in certain peptide hormones such as AVP. However, these findings need further validation using a larger cohort.

Unfortunately, the risk associated with drawing CSF samples at an early age is also present, thus, using a less invasive body fluid is needed as a source for ASD biomarker discovery. For instance, one study tried to investigate whether there is a correlation between AVP concentration in plasma and the CSF. They showed that the blood concentration of AVP positively correlated with the corresponding CSF sample [50]. Many proteomic studies were done using the unbiased MS approach, listed in Table 2. In these studies, serum and plasma were amongst the most common matrix utilized due to their mild invasiveness. The majority of these studies found a differential expression in proteins that are linked to the immune system, lipid metabolism, and platelet function pathways [51,52,53]. Paradoxically, one study failed to confirm the identity of the 8 peaks that were detected in MS and were found to be differentially expressed in ASD subjects [54].

It is important to note that biomarker discoveries are not limited to the brain, CSF, serum, and plasma; other body fluids such as saliva and urine can also be utilized. Two studies utilized saliva samples from ASD patients and age-matched controls [51,58]. Although there were some subtle differences in their methodology, both studies were able to show significant differential expression of proteins related to the immune system pathway. Interestingly, one protein called prolactin-induced protein (PIP) was replicated in both studies mentioned above. The function of PIP is not well-understood; however, it is a promising biomarker for breast cancer [61]. Moreover, PIP is known to bind to CD4+ on T cells; therefore, it may have some immunomodulatory function [62]. In one study, the proteome of urine, first-morning void, was used to search for biomarkers in ASD subjects. They found three proteins were significantly more abundant in ASD compared to controls; these proteins are kininogen 1(KNG1), immunoglobulin heavy constant gamma 1 (IGHG1), and mannan binding lectin serine peptidase (MASP2). Their findings were validated using ELISA, and showed a significant increase in KNG1 in all ASD patients compared to controls [60].

Many studies have used multiplex immunoassays for proteomics biomarker discovery. For instance, a study done on AS, a subtype of ASD wherein individuals present without delays in language development and normal or superior IQ, yet exhibit difficulties in social and communication skills, showed a difference in the plasma proteome of the AS group in comparison to healthy controls in a sex-specific manner [63]. They found an increase in inflammatory cytokines molecules such as interleukin-3 (IL-3), tumor necrosis factor-alpha (TNF-α), and epithelial-derived neutrophil-activating protein (ENA-78) in males, while in females, they observed an increase in androgens, growth, and metabolic pathways such as luteinizing hormone (LH) and insulin [63].

Altered immune response was also evident in ASD, as some studies have shown that cytokines such as TNF-α is significantly expressed in the brain, CSF, and peripheral blood mononuclear cells (PBMCs) of individuals with ASD [64,65,66]. TNF-α is mainly produced by M1 macrophages and it is important for NF-kB activation, which is a transcription factor and an essential regulator of inflammatory genes [67]. The evident increase in TNF-α may suggest a dysregulation in the inflammatory response in ASD. Furthermore, a study found an increased NF-kB binding activity to DNA in the PBMCs of ASD patients [68]. Another essential cytokine is interferon-gamma (IFN-γ), which was found to be elevated in the brain and whole blood of ASD compared to controls [64,69]. Moreover, mothers of individuals who were later diagnosed with autism showed an elevated level of serum IFN-γ, as well as interleukins (IL-4 and IL-5) during mid-gestation [70]. IFN-γ is a pro-inflammatory cytokine that activates CD4+ T-helper 1 (Th1) response [71]. Another pro-inflammatory cytokine interleukin-6 (IL-6) showed a significant production in monocytes of ASD compared to controls when their PBMCs were stimulated with lipopolysaccharide (LPS) in vitro [72]; IL-6 is known to induce T-helper 17 (Th17). Interestingly, when IL-17a was induced in pregnant dam mothers, the offspring exhibited an abnormal cortical development and autistic-phenotypes; even when IL-17a was administrated directly into the fetal brain [73]. Conversely, the administration of anti-IL-17a antibodies in dam mothers during the pregnancy resulted in a reduction in the abnormal behavioral phenotype [73]. These findings highlight the importance of cytokines in discriminating ASD from controls. In addition, they point toward a possible link between immune dysfunction and ASD subtype.

Synucleins have also been studied in the context of ASD. Synucleins are a family of proteins that are abundantly expressed in the presynaptic terminals of the neocortex, cerebellum, thalamus, and striatum [74]. The synuclein family includes α, β, and γ-synucleins (syn). Of a particular interest, the function of α-syn is not well understood. However, α-syn is thought to be involved in vesicle stabilization, synaptic plasticity, and regulates dopamine release [75,76]. α-syn is known to be involved in Parkinson’s disease pathology through an intracellular aggregation process that results in Lewy body formation inside the neurons and eventually, cell death. Although α-syn may play a role in synaptic function, which is thought to be impaired in ASD, there are a limited number of studies on α-syn in the context of ASD. Two studies showed a consistent decrease in α-syn concentration in serum and plasma, respectively [77,78]. However, the latter study was done only on males. On the other hand, β-syn was shown to be higher in ASD patient’s plasma compared to the age-matched controls [78]. Interestingly, autoantibodies against α-syn and other brain proteins were shown to increase in serum of ASD children and their corresponding mothers [79]. This finding may explain the reduced concentration of α-syn in serum/plasma that was evident in the aforementioned studies. Those autoantibodies may mask α-syn epitopes, which could result in α-syn being under-detected.

Furthermore, Tau, which is a major microtubule-associated protein in mature neurons and was known to be hyperphosphorylated in Alzheimer’s disease patients. This hyperphosphorylation causes neurofibrillary tangles and neuronal death [80]. A study showed that Tau concentration decreases in the serum of ASD males [77]. More studies need to be done to elucidate the role of synucleins and Tau in ASD. Moreover, more research needs to be done on more pathogenic forms such as oligomeric and fibril forms of synuclein and Tau.

It is important to note that since ASD risk is traced to around 1000 genetic factors and many environmental factors, it is impractical to assume that individual proteins could be used as a universal biomarker for ASD. Thus, utilizing unbiased methods for proteomics profiling, as discussed in the metabolomic section [81,82], could be more beneficial for patient stratification and biomarker discovery.

## 6. Autoantibodies Biomarkers Suggest a Potential Molecular Sub-Class of ASD

There is growing evidence that supports the involvement of maternal immunity in developing ASD. The notion that maternal autoimmunity may be a cause of ASD in children has been around since the 1970s, as they observed that maternal IgG was present in children’s CSF [83]. A second piece of supporting evidence was by a study cohort that included a large number of subjects (689,196 children). Out of these subjects, 3325 were diagnosed with ASD. The study showed that the risk of ASD increased if the mother had one on the following autoimmune diseases: rheumatoid arthritis, celiac disease, or a family history of diabetes mellitus (DM) type 1 [84]. Comparatively, another systematic review meta-analysis found similar findings, in addition to increased risk of ASD in mothers with hypothyroidism and psoriasis as well [85]. Additionally, when polyclonal antibodies from mothers with children that have ASD were administrated to pregnant mice, the offspring exhibited autistic-like features [86]. Furthermore, monoclonal antibodies against contactin-associated protein-like 2 (Caspr2), which is a membrane protein expressed in the CNS and essential for voltage-gated potassium channels localization in myelinated axons, were generated. Those monoclonal antibodies were successfully able to induce autistic-phenotypes in mouse offspring when exposed to anti-Caspr2 in utero [87]. These early studies laid the groundwork for other studies that aimed to elucidate the link between ASD and autoimmune impairment in the maternal system. Thus, this link could provide a potential biomarker for risk assessment, early intervention, screening, and monitoring for a sub-classification of ASD patients. To understand the role of maternal IgG antibodies in fetal development, some groups tested maternal serum against fetal brain proteins, and they found that maternal IgG antibodies exhibit reactivity against bands at the following molecular weights: 37 kDa, 73 kDa, and 39 kDa [88,89,90], listed in Table 3. Furthermore, the Heuer, Braunschweig, Ashwood, Van de Water, and Campbell study [91], was able to identify the proteins for 37 kDa, 73 kDa, and 39 kDa bands using 2D gel electrophoresis followed by MS. They showed that these bands correspond to lactate dehydrogenase 1 and 2 (LDH1, LDH2), stress-induced phosphoprotein 1 (STIP1), collapsing response mediator protein 1 and 2 (CRMP1, CRMP2), and Y-box binding protein 1 (YBX1); as listed in Table 3. Interestingly, one study tried to investigate the link of promoter allele C (rs1858830) of MET gene with ASD-class that is categorized based on maternal autoantibodies positivity against fetal proteins [92]. The study observed a higher incidence of homozygote MET allele C/C in mothers with a positive fetal protein reactivity and ASD children compared to mothers with typically developed children. Additionally, the homozygote form of MET (rs1858830) C/C alleles were associated with reduced IL-10 concentration, thus it may cause prolonged inflammation [92]. Nevertheless, it is still vague whether the link between maternal autoimmunity to ASD is due to an overlap in susceptibility genes between some autoimmune diseases and ASD or simply a product of IgG infiltration into the fetus’s CNS, hence, interfering with brain development. Solving this dilemma may help in the accurate subclassification of ASD, early diagnosis, and intervention.

## 7. Metabolomics and Gut Microbiome’s Biomarkers in ASD

Metabolic abnormalities are known to be multidimensional in the sense that they cross many pathways such as mitochondrial, oxidative, cholesterol, fatty acid, and neurotransmitters metabolism. Studies suggest a level of dysfunction in these metabolic pathways in ASD patients [94]. Moreover, metabolic pathways are influenced by internal factors such as gene mutations as seen in inborn error of metabolism diseases, and external factors, such as diet, gut microbiome, and exposure to toxins [94]. Some of these metabolites are highlighted in Table 4. The most relevant metabolites to ASD are endocannabinoids, namely anandamide, a fatty acid neurotransmitter and a cannabinoid receptor-1 ligand. The level of anandamide was associated with autism-like features in preclinical animal models including monogenic, fragile-X, and neuroligin 3 models; polygenic, BTBR15, and environmental, valproic acid-induced [95,96,97,98]. As a consequence, two studies found that children with ASD exhibited a significant decrease in plasma and serum anandamide concentration compared to healthy age-matched children [99,100]. Furthermore, another study on rodents showed an alleviation of rodent autism-like traits after rescuing the anandamide pathway by inhibiting its degradation [101]. These findings on the endocannabinoid system do not only help in searching for a biomarker for ASD but also may represent a potential therapeutic system to target. Endocannabinoids are being tested in clinical trials for reducing behavioral problems in ASD subjects (NCT02956226). On the other hand, more studies are shifting toward unbiased methods for metabolite discovery aiming at stratifying patients and looking for sets of biomarkers as therapeutic targets. For instance, a study tried to cluster ASD individuals based on their metabotype focusing on plasma amino acids (AA) profile. They were able to identify a subtype of ASD with an imbalance in AA: branched-chain amino acids (BCAAs). In addition, some amines such as glutamine and ornithine were able to discriminate ASD females in a sex-specific manner [81]. In line with those findings, a clinical trial (NCT02548442) is being tested on ASD subjects aiming to identify biomarkers and stratify ASD based on the metabolomic profile of plasma and urine.

Other interesting metabolomes to be considered are gamma-aminobutyric acid (GABA) and glutamate. These metabolites are important neurotransmitters in the brain. A notable disturbance of the glutamatergic and GABAergic balance in individuals with ASD in comparison to controls has been identified, with a decreased glutamate to GABA ratio, making it a potential area of biomarker exploration for ASD [102]. Although the vast majority of these neurotransmitters are synthesized by the neurons in the brain [103], it is still unclear why their concentration in the peripheral blood is affected. Moreover, short-chain fatty acids (SCFA), produced by intestinal microbiota, was reported to be lower in ASD fecal samples [104]. SCFA act as histone deacetylase inhibitor (HDAC), which is important for glial cell function, and regulate tryptophan 5-hydroxylase 1, which is important for serotonin and tyrosine hydroxylase, a rate-limiting enzyme for the synthesis of dopamine, noradrenaline, and adrenaline [105].

Gut metabolomic studies further point toward a disruption of the normal gut flora and studying the metabolome of body fluids helps in identifying the composition of the gut microbiome. Gut microbiota is known to impact many neurological processes such as blood-brain barrier formation, myelination, and synthesis of neurotransmitters including GABA, dopamine, and others [106]. Those processes are mediated through the microbiota-gut-brain axis, which is a path of bidirectional communication between the CNS and the gut. The link between the gut microbiome and ASD was made because GI disturbances have been frequently detected in ASD [107,108]. One study showed that ASD patients with GI disturbances tend to be more anxious in social situations compared to those with no GI symptoms [109]. The autonomic nervous system that controls gut function is called the enteric nervous system (ENS) and this system shares many structural and functional characteristics with the CNS [110]. Furthermore, a study showed that individuals with ASD that exhibit mutations in the chromodomain helicase DNA binding protein 8 (*CHD8*) gene were reported to have constipation. Furthermore, GI abnormalities were also observed in the zebrafish model with *CHD8* mutations [111]. In addition, germ-free mice that received microbiota transfer from ASD donors showed autistic-like behaviors compared to mice that received a transfer from typically-developed donors [112]. Those mice displayed differences in their metabolome and microbiome profiles evaluated using metagenomic analysis [112]. The evidence that links ASD core symptoms to GI disturbances is strong, and it is being explored in clinical trials. For instance, one clinical trial is trying to test fecal transfer therapy from healthy participants to ASD individuals with GI disorders (NCT03408886).

Gut microbiome diversity is essential for maintaining redundancy and robustness of gut-biochemistry against environmental changes. A study showed that fecal samples taken from ASD subjects showed less gut microbiome diversity and quantity compared to healthy neurotypical controls [113]. In addition, it showed a significant reduction in a genus called *Prevotella* in ASD compared to controls [113]. *Prevotella* is known to colonize the large intestine, and it plays a major role in carbohydrate digestion, which was shown to be disrupted in ASD individuals with GI problems [114]. It has been suggested that clostridium species may exacerbate the symptoms of ASD via exposures to their toxic spores [115]. Another interesting finding is the presence of amino acid phenylaniline metabolites, 3-(3-hydroxyphenyl)-3-hydroxy propionic acid (HPHPA) which is a product of *clostridia* species, in the urine of ASD children [116]. Furthermore, a decrease in the plasma metabolite p-hydroxyphenyllactat, which acts as an antioxidant and is a by-product of *bifidobacteria* and *lactobacillus* [82], has been identified in individuals with ASD. A product called Para-cresol (p-cresol) was shown to be increased in urine [117,118] and feces [119] of ASD patients. p-cresol is produced by *Clostridium difficile* (C. *difficile*) which is a spore-producing anaerobic bacteria, and it has a negative influence on other gut microbiomes especially Gram-negative bacteria [120]. In addition, the severity of ASD symptoms correlated with p-cresol concentration in urine [117]; p-cresol is known to compete with neurotransmitters on the sulfonation process [121]. Moreover, other types of gut microbiome are also affected in ASD, as evidence points toward an elevation in *Firmicutes* to *Bacteroidetes* ratio in feces of ASD patients due to the relative reduction in *Bacteroidetes* [119,122]. Interestingly, a specific species of *Bacteroidetes* called *Bacteroidetes fragilis (B. fragilis)* was shown to improve gut integrity and reduce ASD behavioral symptoms such as anxiety and repetitive behavior when it was administrated in ASD induced mice [123]. The administration of probiotics seems to be beneficial for ASD patients, as it has been explored in clinical trials (NCT02708901), and it showed a positive impact on ASD core symptoms in a subset of ASD patients [124].

Although this field is still in its infancy, it may help in biomarker discovery, and it may add another dimension to the understanding of ASD pathogenesis. These findings suggest that gut-microbiome composition and its metabolome may be used as a clue to understanding how external factors may affect ASD pathogenesis and severity. However, more studies need to be done to delineate the exact mechanisms of how microbiome imbalance contributes to ASD core symptoms.

## 8. Mitochondria Dysfunction in ASD

Mitochondrial dysfunction is linked to ASD. A systematic meta-analysis study showed that the prevalence of mitochondrial diseases in ASD was 4–5%, which is markedly higher than the general population (around 0.01%) [126,127]. Lactate was the first biomarker that was found to be elevated in ASD children’s serum [126,127]. Other mitochondrial biomarkers that were shown to be elevated in children with ASD are AST, pyruvate, and creatine kinase [126,128]. On the other hand, carnitine was shown to decline [126]. Mitochondrial abnormalities such as increased hydrogen peroxide, reduced NADH, as well as mitochondrial DNA (mDNA) over-replication, were observed in lymphocytes isolated from ASD subjects [128]. Using an MS approach for mitochondria biomarker discovery, one study constructed a signature metabolomic pattern that is highly sensitive and specific in predicting ASD patients using plasma samples [82]. Most of these signature molecules identified have been previously reported such as creatinine, fatty acids, 3-aminoisobutyric acid, tricarboxylic acid, and BCAAs [82]. More evidence is pointing towards an association between neurodevelopmental regression (NDR) ASD with mitochondrial dysfunction [129,130]. A recent study has shown that the mitochondrial respiratory rate is elevated in ASD with NDR compared to ASD with no NDR, suggesting a potential subtype of ASD [127].

## 9. Brain Imaging, ERPs, and Eye-Tracking

Biomarker discovery is not confined by the boundaries of molecular investigations. Multiple studies have tried to investigate changes in brain structure and function using brain imaging tools such as magnetic resonance imaging (MRI), computerized tomography (CT) scan, and positron emission tomography (PET) scan. These scanning tools are commonly used to identify different brain features between ASD and typically developed individuals in a hope of improving early diagnosis of ASD. In general, an increase in brain volume was consistently observed in ASD patients compared to healthy controls [131,132]. In addition, the volume of the temporal, frontal lobe, as well as the CSF and lateral ventricles, were found to be increased in ASD subjects [133,134,135]. On the other hand, the corpus callosum, cerebellum, and cerebellar vermin volumes were reduced in ASD subjects [134,136,137]. Paradoxically, despite the fact that the amygdala is known to have a role in fear, social, and communicative activities, which are the core issues in ASD, it was shown to be enlarged only in ASD children not in adults. However, abnormal hippocampus size was noted even during adolescence [138,139].

Shen et al. performed a prospective study that was the first to investigate the extra-axial fluid accumulation in the brain [140]. In their study, they observed fifty-five infants that were divided into two groups. The first group was a high-risk group, which included infants with a family history of ASD (siblings with ASD). The second group was a low-risk group, which included infants with no family history of ASD. They recorded the infant’s brains at different time points between 6–24 months old using MRI. Their results showed that infants who developed ASD later in life had a significant accumulation of extra-axial fluid mainly in the frontal lobes, and larger cerebral volume compared to typically-developed and developmental delay infants [140]. Similarly, another study that used functional connectivity-MRI (fcMRI) imaging on 6-month-old infants with a high risk of ASD showed an accurate prediction of ASD diagnosis by 24 months old, when ASD is known to manifest. They incorporated a machine-learning algorithm to capture the differences in fcMRI images between ASD and non-ASD infants [141].

Utilizing electroencephalographic recordings has been used to explore the ability of ASD individuals to recognize faces and objects by measuring high-density brain event-related potentials (ERPs) by measuring negative central (Nc) and P400 waves as parameters. A study found that ASD individuals failed to show amplitude differences in ERPs of familiar versus unfamiliar faces, while they did show amplitude differences in Nc and P400 in familiar versus unfamiliar objects. In contrast to controls that showed differences in both familiar versus unfamiliar faces and familiar versus unfamiliar objects. This finding suggests impairment in face recognition in ASD individuals [142].

Furthermore, robust findings in ASD brain imaging were noted at an early age (<6 years of age) [143], which may suggest that brain imaging could be a potential tool for early ASD risk assessment. Although MRI imaging is relatively safe, the use of dyes, sedative, or even distress during the procedure may pose a minimal risk to ASD children at that age [144]. As a consequence of that, guardians may be skeptical. Thus, these circumstances call for an urgent need for more age-friendly tools for ASD biomarkers studies or diagnosis.

A new emerging technique for ASD diagnosis is eye-tracking. It is a non-invasive, objective technique in which the subjects are presented with a picture of a human that they need to look at; the device will measure eye gazing time and location [145]. ASD children are known to avoid gazing into the eyes or the center of the face [146]. Likewise, studies done using eye-tracking showed that ASD subjects have significantly lower gazing time at the eyes and the face [146,147,148], and would rather gaze at irrelevant subjects. Interestingly, a preliminary study showed an even more significant difference when ASD subjects (from 4–6 years of age) were asked to look at a speaking face. Their findings suggest that ASD subjects had reduced fixation time at the speaking face compared to the typically developed children. The reduced gazing time was mainly prominent in the areas of eyes, mouth, body, person, face, and outer-person [149]; however, increased gazing time to the mouth was observed in another study [150].

## 10. Lessons from Other Diseases in Precision Medicine

**Figure 2:**
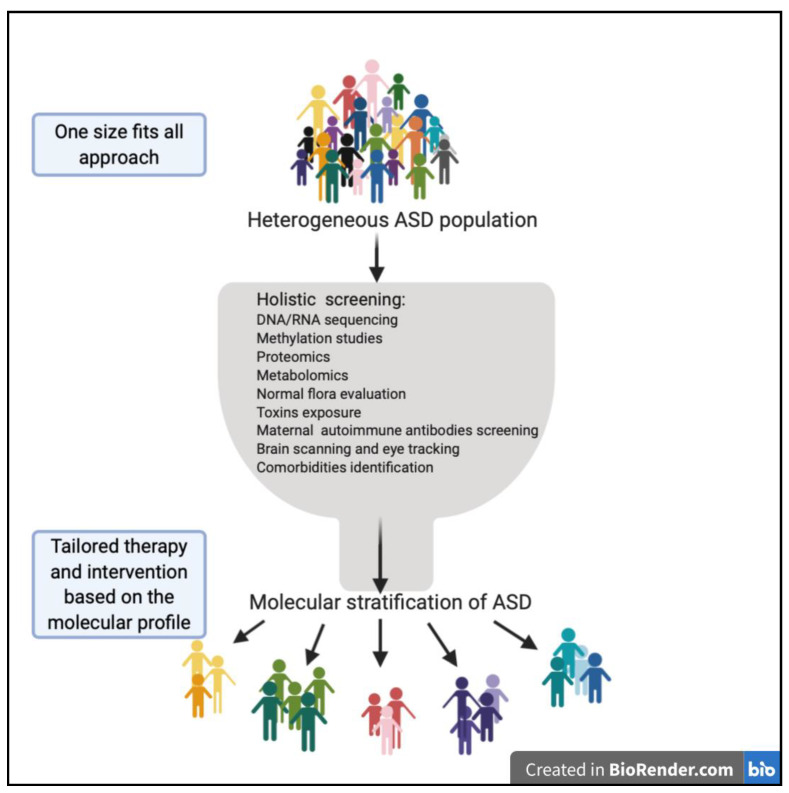
The extreme heterogeneity and complexity of ASD in terms of clinical manifestations, genetic background, and biological changes makes it hard for ASD to fit into a one size fits all treatment and diagnostic approach model; thus, applying a multi-modal approach utilizing modern technologies is a key for proper stratification and achieving tailored therapy that is most fitted to an individual’s condition.

To date, there is a lack of approved biomarkers for ASD screening and diagnosis. Despite the fact that many studies showed promising results in many areas, most of the studies are still in their infancy and lack consistency. This further includes studies that have reached the first and second phase of clinical trials, which later failed to proceed [151]. The causes of these issues are most likely due to the extreme heterogeneity of ASD and the fact that it overlaps with other comorbidities. Hence, models of ASD biomarker discovery, may need to consider its multidimensional complexity. Indeed, after the tremendous improvement in biomedical science technology and sequencing of the human genome, it became possible to use big data to enhance our understanding of ASD by incorporating OMICS into both research and clinics, as illustrated in Figure 2. The ultimate goal of biomarker discovery is implementing biomarkers within clinical settings to provide ASD risk assessment, screening, diagnosis, monitoring, and stratifications for better therapeutic strategies. Biomarkers for early diagnosis and stratification are desperately needed for ASD, especially for early diagnosis, hence, early intervention, an essential key for better outcomes.

Likewise, it is important to develop biomarkers to stratify patients for therapeutic purposes so that trials could be more effective. For instance, if a particular drug seems to show a potential link to a biomarker, biomarker-targeted trials can be initiated using that drug (i.e., GABAergic biomarkers are used to monitor drugs that target the GABAergic system, and clinical trials are trying to target the GABAergic system as a therapeutic strategy for ASD, such as NCT03678129 clinical trial). Stratification of biomarkers in therapeutics is reviewed in [152].

A well-known example of utilizing precision medicine in patient stratification is cystic fibrosis (CF). CF is an autosomal recessive disease that is caused by mutations in the cystic fibrosis transmembrane conductance regulator (CFTR) gene. This gene can carry more than 1000 mutations and more than 100 are known to be pathogenic [153]. Understanding the effect of these mutations helped in stratifying individuals into six classes based on mutations and the defects they cause. Moreover, this classification helped in devising an optimal therapy plan for the patients. Additionally, although CF is a monogenetic disease, symptoms vary in severity even if patients bear the same genetic mutation, this phenomenon can be explained by exposure to environmental factors and modifier genes that contribute to the severity of CF [153]. Even though ASD and CF differ in many ways, applying a similar concept that utilizes multi-modal molecular stratification may help in tailoring the intervention strategies in such a way that is more suitable to the patients.

Another worthy example of the importance of patient stratification is anemia. Anemia is a blood disease caused by low hemoglobin concentration, with a prevalence of 24.8% worldwide as estimated by the World Health Organization (WHO) [154]. Hemoglobin is a characteristic protein expressed in red blood cells (RBC) and is important for gas exchange. There are many subtypes of anemia that are sub-classified based on multiple parameters, such as RBC microscopic morphology, mean corpuscular volume (MCV), and hemoglobin concentration (MCHC), iron levels, protein electrophoresis, sequencing as well as vitamin B12 and folate concentrations, and other parameters [155]. Different sub-classes of anemia may share many clinical symptoms; however, they differ at multiple cellular and molecular levels, and they have different treatment strategies. Similar examples of using biomarkers for stratification and targeted therapy were seen in cancer such as non-small cell lung cancer (NSCLC), breast cancer, colorectal cancer [156,157]. Genomics testing such as testing for *CYP2C9* and *VKORC1* variants are used for warfarin dosage determination for patients with cardiovascular diseases [158]. With regard to the CNS, neurons are structurally complicated compared to blood and other tissues, and the brain tissue is inaccessible, which makes it hard to study during human development. Nevertheless, those abovementioned examples show that heterogeneity exists in many diseases, and patient stratification is the solution for understanding pathogenesis and optimizing therapies.

With respect to precision medicine in ASD, a recent study highlighted the possibility of using a multi-modal approach for patient stratification. In this study, large WES data along with spatiotemporal expression of genes during brain development was used to identify variants that are deleterious, ASD-segregated, developmentally co-regulated, and sex-specific. They found dyslipidemia as a common theme in a subset of non-syndromic ASD individuals and their findings were validated using massive electrical health records (EHR) and medical claims [159].

## 11. Contemporary Challenges and Future Directions

As the pathogenesis and the etiology of ASD are still not well-understood, OMICS multi-modal approaches could pave the way towards elucidating the etiology of ASD. Given the urgent need for an early diagnostic biomarker, researchers have been investigating all body fluids such as CSF, blood, saliva, urine, and stool looking for a possible set of biomarkers. Although there are many promising findings, it is still early to implement these findings for the early diagnosis of ASD. Alternatively, using these findings to thoroughly stratify ASD individuals based on their molecular profile could be a possible approach. Additionally, there are factors that need further consideration, these factors may contribute to the inconsistencies and the lack of replication between biomarker discovery studies. First, the definition of ASD and the method of diagnosis varied between studies. Additionally, comorbidities are not uncommon among ASD individuals. Subsequently, including these subjects could affect the accuracy of the findings, especially at the transcriptomic, proteomic, and metabolomic levels. The second important point is the consideration of therapies and medications that may have been undergone by ASD individuals while being involved in studies; these interventions may hinder or modify possible findings. Thirdly, the age range of the groups that participate in ASD biomarker studies is crucial, as reviewed above, mainly because the brain continues to change dramatically during childhood and adolescence. Furthermore, gender should also be considered during biomarker discovery studies. Finally, at the technical level, the method of sample processing is important because it is one of the main factors that contribute to the variability between the studies, not to mention the importance of selecting proper tissues/matrices mainly for proteomics, transcriptomics, and metabolomic studies.

Biomarker discovery has the potential to tailor therapeutic interventions to fit individualized conditions in order to receive maximum benefits. However, the question is why has it not been the case for ASD? Why have we not identified a robust set of ASD biomarkers that can be implemented in a clinical setting? Nevertheless, as biomedical technologies evolve and more discoveries on ASD pathogenesis surface, the more likely we are to utilize this knowledge for one’s benefit. Furthermore, at this stage, having a tunneled vision at a specific aspect for biomarker discovery may not give the best answers, but rather, a thorough study on case-by-case bases and collecting data as much as possible at multiple levels on ASD may help unravel the answer.

## Figures and Tables

**Table 1:** Genes associated with autism spectrum disorder (ASD).

Gene Name	Function	Reference
Astrotactin- 2 (*ASTN2*)	Neuronal adhesion molecule has a role in glial migration.	[19]
Contactin 4 (*CNTN4*)	Neuronal maintenance and plasticity.	[20]
F-Box Protein- 40 (*FBXO40*)	Ubiquitin-protein transferase activity.	[19]
*FMRP translational regulator-1 (FMR1)*	mRNA trafficking from the nucleus to the cytoplasm. Synaptic plasticity.	[21]
Potassium voltage-gated channel subfamily Q member 2 (*KCNQ2*)	Transports potassium ions inside and outside the cells.	[22]
*lysine methyltransferase 2E (KMT2E)*	Regulates gene transcription.	[22]
*Mono-ADP-Ribosylhydrolase (MACROD2)*	Remove ADP-ribose from mono-ADP-ribosylated proteins.	[22]
*Methyl CpG binding protein- 2 (MeCP2)*	Chromosomal protein that binds to methylated DNA, it binds to single methy-CpG pairs.	[21]
Neuronal growth regulator- 1 (*NEGR1*)	Regulates synapses formation in the hippocampus.	[22]
Neuroligin-1 (*NLGN1*)	Synaptic functions and transmission.	[19]
Neurexin- 1 (*NRXN1*)	Binds neuroligins and formation of synaptic contacts.	[23]
*Parkin (PARK2)*	Part of protease complex multiprotein that guides to proteasomal degradation.	[19]
Polypyrimidine tract binding protein-2 (*PTBP2*)	Control assembly of splicing- regulatory proteins and important for alternative splicing in early development.	[22]
Ring finger and WD domain 2 (*RFWD2*) Also, known as *COP1*	Mediates ubiquitination and substrate protein degradation.	[19]
SH3 and multiple ankyrin repeat domains protein-3 (*SHANK3*)	Scaffold protein of the postsynaptic density.	[24]
Tuberous sclerosis complex (*TSC1* and *TSC2*)	Tumor suppressor gene that activate GTPase activating protein tuberin.	[25]
*ubiquitin protein ligase E3A* (*UBE3A*)	E3 ubiquitin-protein ligase.	[19]

**Table 2:** Proteomics studies on ASD.

Sample Type	Detection Method	Proteins Identified	Function	Reference
Serum	Tricine-PAGE LC-MS/MS	ApoA1, ApoA4, PON1.	Cholesterol metabolism Oxidative damage	[55]
Serum	multiplex immunoassay LC-MS	Immune assays Females: ADIPO, APOA1, IgA Males: IL-12p70, IL-16, TF, TNF-alpha, BMP6, CTGF, ICAM1. Both: CHGA, EPO, IL-3, TENA, PAP, SHBG. LC-MS Females: APOC2, APOE, ARMC3, CLC4K, FETUB, GLCE, MRRP1, PTPA, RN149, TLE1, TRIPB, ZC3HE. Males: RGPD4.	Cholesterol metabolism and transport Inflammation Androgens	[56]
Serum	MALDI-TOF MS	SERPINA5, PF4, FABP1, APOC1, AFP, CPB2, TAAR6, FGA.	Platelets and coagulation functions Cholesterol metabolism	[53]
Serum	MALDI-TOF MS	Eight peaks (Unidentified) 6.42 kDa, 7.75 kDa, 9.27 kDa, 3.88 kDa, 6.62 kDa, 4.08 kDa, 4.64 kDa, 4.20 kDa.	-	[54]
Serum	LC-ESI-MS on TOF	FHR1, FN1, *C1q,* B-100.	Cholesterol metabolism Complement system	[57]
Saliva	Nano LC-MS	PIP, LTF, IGKC, IGHG1, IGLC2, NE, pIgR, DMBT1.	Immune system	[51]
Saliva	2D-PAGE Nano LC-MS/MS	AMY1A, *CBP, P532,* TF, ZAG, ZG16, CST5, PLG, FRAT1, KIF14, ITGA 6, GRTP1, hPSP, PIP, MUC16.	Lipid and cholesterol metabolism Immune system Oxidative damage	[58]
Saliva	LC-MS/MS	Hypo-phosphorylation of STATH, HTN1, aPRP.	-	[59]
Urine	2D-PAGE MALDI-TOF MS	KNG-1, MASP2, IGHG1.	Immune system Coagulation	[60]

**Table 3:** Autoantibodies studies on ASD.

Observed Autoantibodies Reactivities	Molecular Weight	Samples Used	Reference
Human fetal brain proteins Human adult brain proteins	73 kDa and 37 kD -	Mother’s serum of: ASD vs. non-ASD (DD) * vs. TD *	[90]
Human fetal brain proteins	39 kDa, 39 kDa and 73 kDa	Mother’s plasma of: AU * vs. ASD vs. DD * vs. TD *	[88]
Human fetal brain proteins Human adult brain proteins Rodent embryo brain proteins Rodent adult brain proteins	36 kDa, 39 kDa and 61 kDa caudate at 155 kDa and BA9 at 63 kDa 36 kDa and 73 kDa 27 kDa	Mother’s serum of: ASD vs. controls	[93]
LDH 1, LDH2, STIP, CRMP1, CRMP2, and YBX1	37 kDa, 39 kDa, 48 kDa, 62 kDa and 68 kDa	Mother’s plasma of: ASD vs. controls	[91]

* AU: full autism; DD: developmental delay; TD: typically developed child.

**Table 4:** Metabolomic biomarkers in ASD.

Metabolite	Sample Type	Method of Detection	Effect/Function	Reference
Anandamide (decrease)	Serum/Plasma	LC-MS/MS	Endocannabinoid signaling	[99,100]
HPHPA (increase)	Urine	GC-MS	A by-product of *clostridium* species and a probable tyrosine analog of m-tyrosine (3-hydroxyphenylalanine), that may depletes brain catecholamines.	[116]
p-hydroxyphenyllactat (decrease)	Plasma	LC-HRMS	A by-product of *bifidobacteria* and *lactobacillus,* act as an anti-oxidant	[82]
p-cresol (increase)	Urine Feces	HPLC-UV GC-MS/SPME	Competes with neurotransmitters on the sulfonation process and disturbs gut microbiome	[117,118,119]
GABA (increase)	Plasma	ELISA	Neurotransmitter	[102,125]
Glutamic acid (increase)	Plasma	LC-HRMS	Amino acid	[82]
SCFA (decrease)	Feces	FID-GC	Regulate tryptophan 5-hydroxylase 1 which is important for serotonin, dopamine, adrenaline and nor adrenaline production.	[104]
Lactate (increase)	Serum	ELISA and colorimetric assays	Energy metabolism	[125]
Pyruvate (increase)	Serum	ELISA and colorimetric assays	Energy metabolism	[125]
5-Aminovaleric acid (increase)	Plasma	LC-HRMS	Lysine degradation product and week inhibitor of coagulation	[82]
DHEA-sulfate (increase)	Plasma	LC-HRMS	Sex-hormone	[82]

## Data Availability

Not applicable.

## Abbreviations

ADHD	Attention deficit hyperactivity disorder
ADOS	Autism Diagnostic Observation Schedule
AGE	Glycation end products
ASD	Autism spectrum disorder
BA10	Brodmann area 10
CARS	Childhood Autism Rating Scale
CGH	Comprehensive genomic hybridization
CNV	Copy number variant
CSF	Cerebrospinal fluid
CVD	Cardiovascular diseases
DD	Developmental delay
ERP	Event-related potentials
GI	Gastrointestinal
T2DM	Diabetes mellitus type 2
DSM-5	Diagnostic and statistical manual of mental disorders- 5
DZ	Dizygotic
fcMRI	Functional connectivity magnetic resonance imaging
FXS	Fragile X syndrome
GC	Gas chromatography
GWAS	Genome wide association studies
iASD	Idiopathic Autism spectrum disorder
ICD-10	International Classification of Disease 10
LC	Liquid chromatography
MALDI-TOF	Matrix-Assisted Laser Desorption Ionization Time-of-Flight
miRNA	microRNA
MS	Mass spectrometry
mtDNA	mitochondrial DNA
MZ	Monozygotic
ncRNA	non-coding RNA
NGS	Next generation sequencing
NDR	Neurodevelopmental regression
PDD-NOS	Pervasive developmental disorder not otherwise specified
PTM	Post-translational modifications
rRNA	Ribosomal RNA
RT-PCR	Reverse transcriptase-polymerase chain reaction
snoRNA	Small nucleolar RNA
snRNA	Small nuclear RNA
SNV	Single nucleotide variant
SV	Structural variants
TD	Typically developed
TLDA	TaqMan low-density array
TSC	Tuberous sclerosis complex
WES	Whole-exome sequencing
WGS	Whole-genome sequencing
